# Silent volumetric multi-contrast 7 Tesla MRI of ocular tumors using Zero Echo Time imaging

**DOI:** 10.1371/journal.pone.0222573

**Published:** 2019-09-16

**Authors:** Jan-Willem M. Beenakker, Joep Wezel, Jan Groen, Andrew G. Webb, Peter Börnert

**Affiliations:** 1 Department of Radiology, Leiden University Medical Center, Leiden, the Netherlands; 2 Department of Ophthalmology, Leiden University Medical Center, Leiden, the Netherlands; 3 Philips Healthcare, Best, the Netherlands; 4 Philips Research Laboratories, Hamburg, Germany; Wayne State University, UNITED STATES

## Abstract

Magnetic Resonance Imaging (MRI) has become a valuable imaging modality in ophthalmology, especially for the diagnosis and treatment planning of patients with uveal melanoma, the most common primary intra-ocular tumor. We aim to develop and evaluate the value of silent Zero Echo Time (ZTE) MRI to image patients with ocular tumors at 7Tesla. Therefore, ZTE and different types of magnetization-prepared ZTE (FLAIR, SPIR, T2 and Saturation recovery), have been developed. After an initial validation with 7 healthy subjects, nine patients with an eye tumor have been evaluated. The ZTE scans were compared to their Cartesian equivalent in terms of contrast, motion-sensitivity, diagnostic quality and patient comfort. All volunteers and especially the patients reported a more comfortable experience during the ZTE scans, which had at least a 10 dB lower sound pressure. The image contrast in the native ZTE was poor, but in the different magnetization-prepared ZTE, the eye lens, cornea and retina were clearly discriminated. Overall the T2-prepared scan yielded the best contrast, especially between tumor and healthy tissue, and proved to be robust against eye motion. Although the intrinsic 3D nature of the ZTE-technique provides an accurate analysis of the tumor morphology, the quality of the ZTE-images is lower than their Cartesian equivalent. In conclusion, the quality of magnetization-prepared ZTE images is sufficient to assess the 3D tumor morphology, but insufficient for more detailed evaluations. As such this technique can be an option for patients who cannot comply with the sound-levels of Cartesian scans, but for other patients the conventional Cartesian scans offer a better image quality.

## Introduction

In recent years, MRI has become a valuable imaging modality in ophthalmology due to its ability to non-invasively image parts of the eye which are not accessible by conventional optical techniques [1–5]. One of the key applications of ocular MRI is providing accurate three-dimensional geometric measures on the location, shape and size of tumors. This is critical for patient treatment, which consists either of eye-preserving radiotherapy or total eye removal. Accurate size measurements require very high spatial resolution, and so ocular imaging can benefit from high and ultra-high field MRI due to the increased SNR available. A recent high field study showed the clinical potential of ocular MRI as it provides a more accurate geometrical description of tumors and surrounding tissues compared to conventional ultrasound[4].

However, one of the main challenges in MRI is motion and in ophthalmology in particular: eye-motion. This results in significant image artifacts, which can mask extrascleral extension of the tumor. Different strategies have been developed to reduce the amount of motion artefacts in ocular MRI scans, which can last several minutes. Among them are cued-blinking protocols, which incorporate regular pauses in the scan during which the subject is allowed to blink[6,7]. These strategies, which rely on active patient cooperation, are able to resolve most of the motion-artifacts in eye imaging, but make the protocol and setup also more complicated. In addition, according to our experience, the sudden increase of the gradient-induced acoustic noise at the beginning of each new acquisition-block after a blinking pause tends to provoke subconscious eye-blinks. Therefore, low acoustic noise MRI sequences would have many advantages for this kind of application.

Zero echo time (ZTE) imaging[8,9], a 3D radial FID-sampling technique, which has been shown to be able to visualize highly ordered, fast T2-relaxing tissues at isotropic spatial resolution, could be a very interesting candidate especially due to its low acoustic noise. This feature is a consequence of the fact that in ZTE RF excitation and signal sampling take place in the presence of the same gradient. This gradient is active during the entire 3D experiment at a fixed strength, changing its direction only very slightly from shot to shot. This feature can reduce the acoustic noise to the whisper level. However, ZTE is often restricted to low excitation flip angles [10], removing any potential T1-contrast, and very short effective echo times (TE), which is actually the key feature of ZTE. Therefore, the native ZTE image contrast is rather poor, because all species contribute to the image as there is no time for most of the contrast mechanisms to evolve. To mitigate this problem longitudinal magnetization preparation is proposed to address a number of potentially useful contrasts[11,12].

In this study we evaluated the use of 3D, ultra-high field, magnetization-prepared ZTE imaging to visualize the eye for two main reasons. First, ZTE sequences have a very low acoustic noise level, which significantly increases patient comfort and decreases the involuntary blink reflexes. Secondly, the inherent isotropic spatial resolution of this 3D sequence supports reformatting in all directions retrospectively, which is advantageous in terms of the highly irregular shape of tumors. Often multiple cuts in different orientations are required to assess the optimal treatment and therefore a isotropic 3D sequence significantly helps to simplify the scanning workflow. After outlining the basic features of the ZTE sequence, aspects of magnetization preparation for eye imaging will be discussed together with results of an initial patient study performed at ultra-high field strength.

## Materials and methods

### Basic ZTE methodology and contrast manipulation

**Fig 1:**
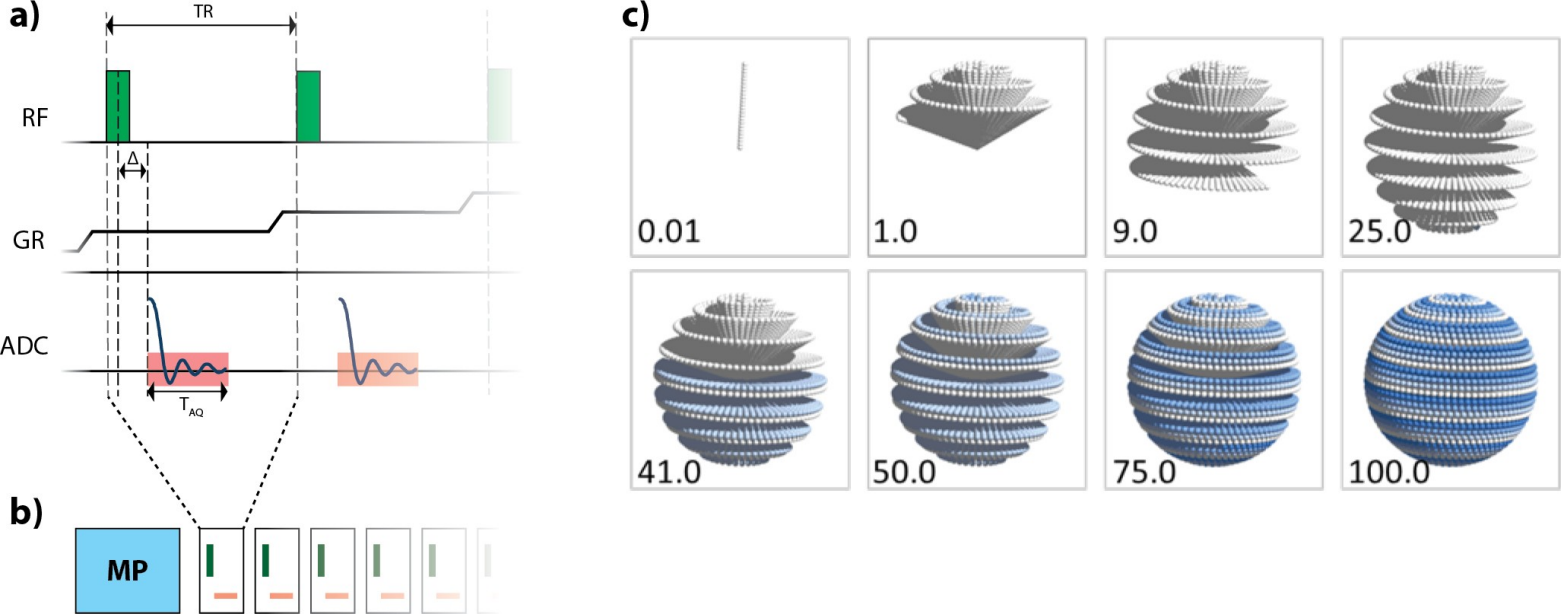
Magnetization-prepared ZTE scheme. (a) The basic ZTE acquisition sequence. After a non-selective excitation pulse, applied in the presence of the read-out gradient (GR), sampling takes place after a system specific dead time Δ, to switch from the transmit to the receive state. (b) After appropriate Mz magnetization preparation (MP) a number of ZTE profiles are measured in a train spiraling further along the given trajectory path. Note, as illustrated schematically in (a), that the read-out gradient is on all the time, changing only slightly its direction from shot to shot. (c) Shows the temporal order of the individual ZTE spokes in the 3D radial k-space. The numbers indicate the scanned k-space percentage.

The ZTE sequence was integrated into the existing 7T MRI system (Philips Achieva, Cleveland, USA) software without any hardware changes. In the current implementation ZTE imaging is performed with a short block excitation RF pulse (12.8μs duration), applied in the presence of a read gradient which slightly changes direction each TR (Fig 1). The individual spokes of this 3D radial trajectory follow a spiral trajectory running from the north to south pole on the surface of a unit sphere[13]. In the current implementation 20 of these north-to-south-pole runs are used to sample k-space, which is schematically illustrated in Fig 1. Due to the short RF pulse and maximum RF transmit amplitude restrictions (B_1_^+^) of the quadrature RF transmit coil (Nova Medical), the flip angle is limited to 3°. Signal reception is performed using a dedicated proton eye receive coil (diameter 40 mm), integrated into special goggles to allow visual communication with the patient[4]. This coil was designed to increase the SNR and to localize the area the MR signal to the eye, facilitating small field-of-view (FOV) imaging.

In a pre-study, different types of magnetization-prepared sequences were evaluated to determine the optimal contrast to discriminate the tumor from the surrounding structures. These scans were performed at an isotropic spatial resolution (1.0 mm)^3^ to limit the total scanning time. The basic ZTE imaging parameters were chosen as follows: FOV of (120 mm)^3^, TR 2.0 ms, 100% angular sampling, resulting in a total scanning time 0:57 minutes for a native ZTE with proton density (PD) contrast. Due to the length of the RF pulse and finite switching time from transmit to receive state on the unmodified MR system, the TE was fixed to 67μs. Extra data sampling at a reduced read gradient strength was used to fill the missing samples in the centre of k-space [14,15]. Apart from native ZTE free induction decay imaging, segmented magnetization-prepared approaches were investigated, listed in Table 1. First, fluid-attenuated inversion recovery (FLAIR) contrast was realized by embedding the segmented ZTE acquisition into an inversion recovery sequence. The corresponding pre-pulse was repeated every 3 s to allow for T_1_ relaxation. A similar contrast to its Cartesian equivalent was achieved by setting the same inversion time of 1280 ms. Second, T_2_-weighting was achieved by applying a T_2_-preparation module[16] with two adiabatic 180° RF refocusing pulses to minimize B_1_^+^ effects. Preliminary evaluations showed that an echo time of 30 ms resulted in sufficient T_2_-weighting to differentiate the sclera from the surrounding structures. Third, spectral partial inversion recovery (SPIR) was employed for fat suppression, while finally, a saturation recovery contrast (SAT) was used, applying a 90° saturation RF pulse followed by a ZTE sampling block. The sampling time per shot and shot intervals, listed in Table 1, were chosen such to optimize the trade-off between signal-to-noise (longer delay between shots), contrast wash-out (less spokes per shot) and total scan time. Based on the results of the pre-study, a high, (0.8mm)^3^, resolution version of the T_2_-prepared ZTE sequence was developed. This acquisition time of this scan was approximately 3 minutes, which is similar to its Cartesian equivalent, allowing for a clinically relevant comparison.

**Table 1:** ZTE imaging parameters.

Contrast	Prepulse	Acquisition resolution	Sampling time	Echo train length	Shot interval	Scan time
Native ZTE	-	1.0 mm	-	-	-	0:57 min
T1-weighted	FLAIR with 1280ms delay	1.0 mm	480ms	240	3s	6:02 min
T2-weighted	T2-prep; TE: 30ms	1.0 mm	360ms	180	900ms	1:54 min
Fat suppressed	SPIR pulse	1.0 mm	100ms	50	124ms	1:01 min
Saturation	90° Sat. pulse	1.0 mm	361ms	180	900ms	1:50 min
T2-weighted, higher resolution	T2-prep; TE: 30ms	0.8 mm	414ms	207	900ms	2:47 min

Main scan parameters for the ZTE scans. The FOV for the (1.0mm)^3^ scans was (120mm)^3^ and was increased to (140mm)^3^ for the higher resolution (0.8mm)^3^ scan. The bandwidth of the 1.0mm^3^ and (0.8mm)^3^ scan were 425 Hz and 336 Hz respectively.

To compare the sound level of the ZTE scans with their Cartesian equivalent[4], the sound pressure was measured in the scanner room with a Voltcraft SL-100 Decibel meter (Germany).

### In-vivo studies

Before studying the performance of the magnetization prepared ZTE in patients, three in-vivo tests were performed in a small volunteer cohort (7 volunteers, ages 25–35). Written consent was obtained according to the rules of the institution.

### Volunteer study on image contrast

In the first study the basic image quality of the magnetization prepared ZTE was investigated. This helps to judge the basic potential of the ZTE regarding the degree of contrast manipulations compared to selected Cartesian sequences specifically tailored previously for high definition eye tumor imaging[4,7]. In previous eye tumor studies, the FLAIR contrast in particular has been found to be essential for accurate clinical diagnosis. The parameters used for the Cartesian 3D FLAIR, listed in Table 2, included a spatial resolution 0.5×0.5×1.0 mm^3^ a TR/TE/FA of 5.6 ms/2.9 ms/7° respectively, and an inversion delay of 1280 ms using low-high k-space sampling for 519 ms. The images were visually compared to the ZTE results by two independent readers (general MRI expert with over 30 years experience (PB), ocular MRI expert with 7 years experience (JWB)).

**Table 2:** Cartesian iymaging parameters.

Contrast	Scan type	Acquisition resolution	TR/TE/FA/ Echo train length	Bandwidth	Shot interval	Scan time
T1-weighted	3D Gradient echo; FLAIR with 1280ms delay	0.5x0.5x1.0 mm^3^	5.6ms/2.9ms/7°/92	467 Hz	3s	2:53 min
T2-weighted	3D TSE	(0.6 mm)^3^	194ms/2500ms/90°/120	980 Hz		2:53 min

Main scan parameters for the Cartesian scans, with both a FOV of 40x46x38mm^3^.

### Study to judge magnetization transfer effects

The effects of magnetization transfer (MT) [17] in magnetization-prepared ZTE applications were briefly investigated too. The chemical shift selective pre-saturation RF pulse, used in SPIR, which is applied off resonant at the fat frequency, can have an effect on the water signal via MT. This effect was investigated by applying the fat selective RF pre-pulse symmetrically on the other side of the water resonance.

### Sensitivity of the ZTE sequence to motion

The third study addresses the sensitivity of ZTE to eye-motion. For Cartesian magnetization prepared imaging, cued blinking approaches are used to freeze potential eye motion. For this purpose, the patient gets visually instructions to focus (eye opened) on a fixation cross on a projection screen during MR data acquisition. Every three seconds, an interval without data sampling is added to the scan during which the fixation cross automatically changes into a red circle by which the subject is signaled to blink shortly. Previous studies [6,7,18] have shown that this cued blinking approach not only effectively prevents eye-blink related artifacts, but also increases the patient comfort compared to focus-only paradigms. We studied whether this sophisticated kind of motion freezing approach is needed in ZTE applications as well. The T_2_ prepared ZTE approach was chosen and modified to include the above mentioned 3 seconds blinking time, resulting in a slightly prolonged acquisition time (2:53 min) compared to the basic T_2_ prepared ZTE approach (1:54 min). This scan was performed twice. In the first run the volunteer was asked to close the eyes for the entire scan and relax. No further instructions were given during the scan. Before the second run, the subject was instructed to follow the cued-blinking paradigm and the visual instructions were enabled. The uninstructed scan was always performed before the instructed scan, as after one scan with visual blink instructions, subjects tend to still synchronize their blinking with the pauses in the MR sound, regardless of the disabled visual cues. The image quality of both runs was compared visually.

### Patient study

The ZTE protocols described above (native, FLAIR, T2-weigthed and fat-suppressed) were used in a small patient pilot study approved by the local ethical committee, with written consent obtained. Additional ZTE-scanning was performed during a normal eye tumor diagnostic session based on Cartesian scanning, which consisted of FLAIR, T_1_ and T_2_ scans with a cued-blinking paradigm. The ZTE scanning was performed for a maximum-possible total scanning time of 10 minutes, in order to not exceed the maximum time in the magnet constraint.

First four patients (all male, age: 61–69 years) were scanned with the lower resolution ZTE-scans to assess the optimal contrast for imaging ocular tumors and to confirm volunteer findings. Due to the scanning time constraints the actual ZTE scan selection was randomized among the patients. In all patients the fat-suppressed and T_2_-weigthed scans were used. In one patient the native ZTE and FLAIR were performed too. In two other patients, the native ZTE scan was evaluated, while in one patient the FLAIR variant was used.

Subsequently, five additional patients (3 male, 2 female, age 29–80 years) were scanned with the high-resolution T_2_-prepared ZTE scan, to assess the clinical potential of this ZTE approach for ocular tumors. In these scans and their Cartesian equivalent (3D TSE; TR/TE: 2500ms/194ms, refocusing angle: 35°, SPIR fat-suppression, resolution: (0.6 mm)^3^, FOV:40x47x38mm^3^, scan time: 3 minutes) the tumor thickness, a three-dimensional measure which determines the optimal treatment modality[4], was measured by an experienced reader to assess the clinical potential of the ZTE scans for therapy planning. Furthermore, the overall diagnostic quality of both scans was qualitatively evaluated, in terms of sharpness of anatomical boundaries and visibility of the sclera, by two independent observers (PB, JWB).

## Results

### Volunteer study on image contrast

**Fig 2:**
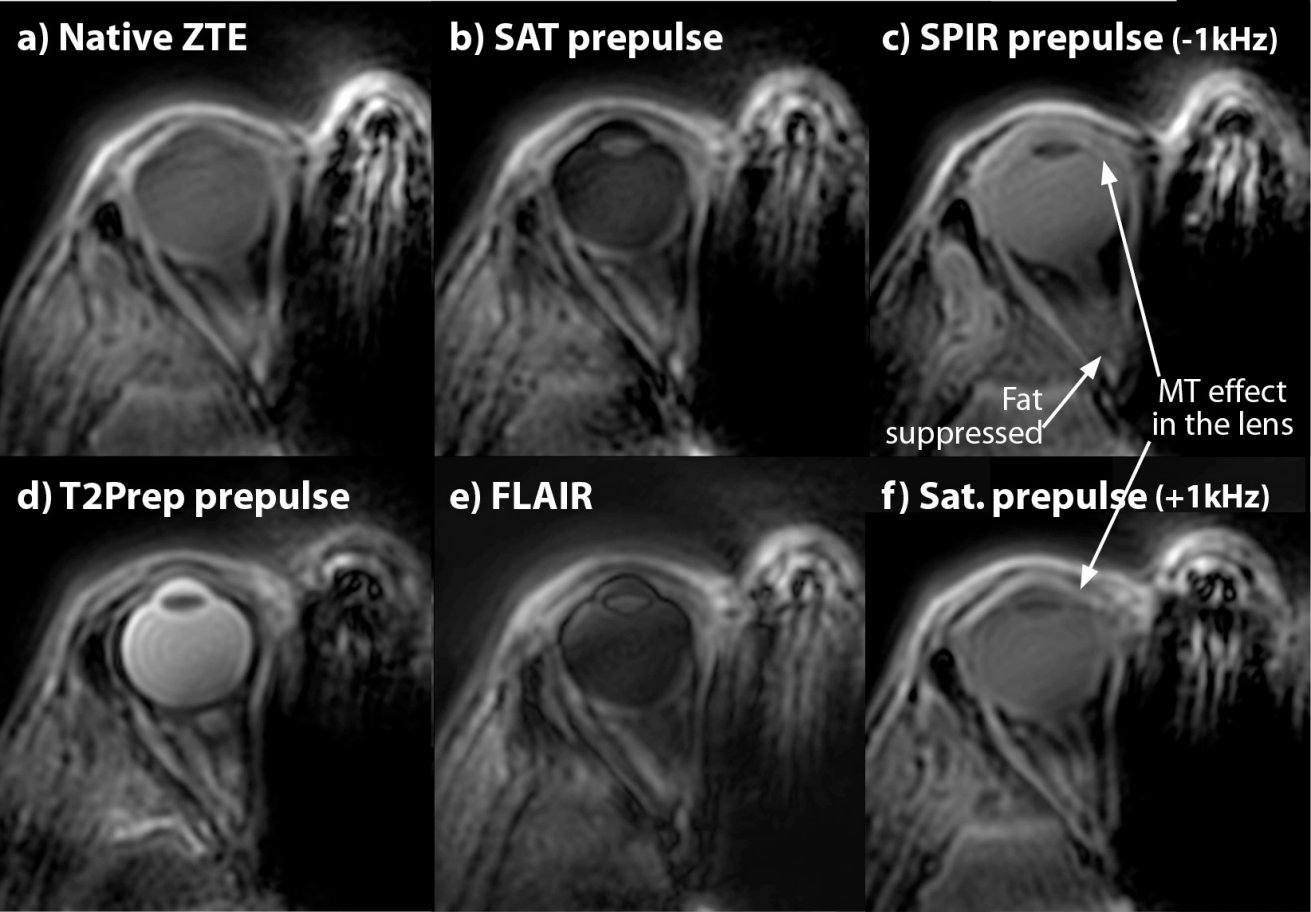
Magnetization-prepared eye ZTE imaging. One selected reformatted slice from the 3D data sets of a healthy volunteer. (a) native ZTE. The eye and parts of the bony structures (especially in the nose area) are visible. Due to the short TE and the low flip angle, the overall contrast is low. (b) A SAT pre-pulse inverts the contrast between eyeball and lens. (c) Fat suppressed (SPIR) ZTE showing improved contrast in the eye and around the nerve and the lens is now visible. (d) T_2_-prepared shows the highest contrast of all sequences. (e) FLAIR-ZTE contrast has low signal from the vitreous body and high signal from the lens and sclera. (f) ZTE image like in (c) but applying the fat saturation RF pulse symmetrically on the other side of the water resonance. This shows no fat suppression but slight contrast in the lens, indicating MTC effect caused by saturating bound water protons.

Fig 2 shows selected transverse reformats of 3D isotropic ZTE data using different magnetization preparations measured in a healthy volunteer. Those images are representative for the entire volunteer cohort. As expected the native ZTE contrast is poor and mainly proton density weighted, although some T_1_ contrast can be seen between the sclera and the vitreous body, due to the relative long T_1_ of the vitreous body of approximately 6 s[19]. The SAT- and FLAIR-prepared ZTE scans, shown in (Fig 2B and 2E), both result in images in which the signal from the vitreous body is suppressed due to its long T_1_. The lens, cornea and retina can clearly be discriminated. Fat suppressed SPIR-ZTE (Fig 2(C)) allows for the clear identification of the different ocular muscles and optic nerve, which are surrounded by the fat. A hypointense signal is associated with the crystalline lens. Finally, the T_2_-prepared ZTE (Fig 2(D)) results in a typical T_2-_weighted image in which the vitreous body and anterior chamber appear bright, while the sclera and lens, due to their relatively short T_2_[20] have a relatively low signal intensity.

Note that the bony structures contribute to the signal in all scans, regardless of the magnetization preparation applied, since the very short T_2_ components are not very much affected by the pre-pulses. This is caused by the fast T_2_-induced loss of coherence in theses tissue types, which takes place during the applied RF in the magnetization preparations[21].

### Study to judge magnetization transfer effects

Magnetization transfer effects caused by the contrast preparation pre-pulses are already visible in the volunteer data shown in (Fig 2C and 2F). The fat suppression or off-resonant excitation on the opposite side of the water spectrum also influences the contrast of non-fatty tissues like the lens. However, in this ophthalmic ZTE application this additional contrast mechanism is beneficial to further improve tissue differentiation.

### Sensitivity of the ZTE sequence to motion

**Fig 3:**
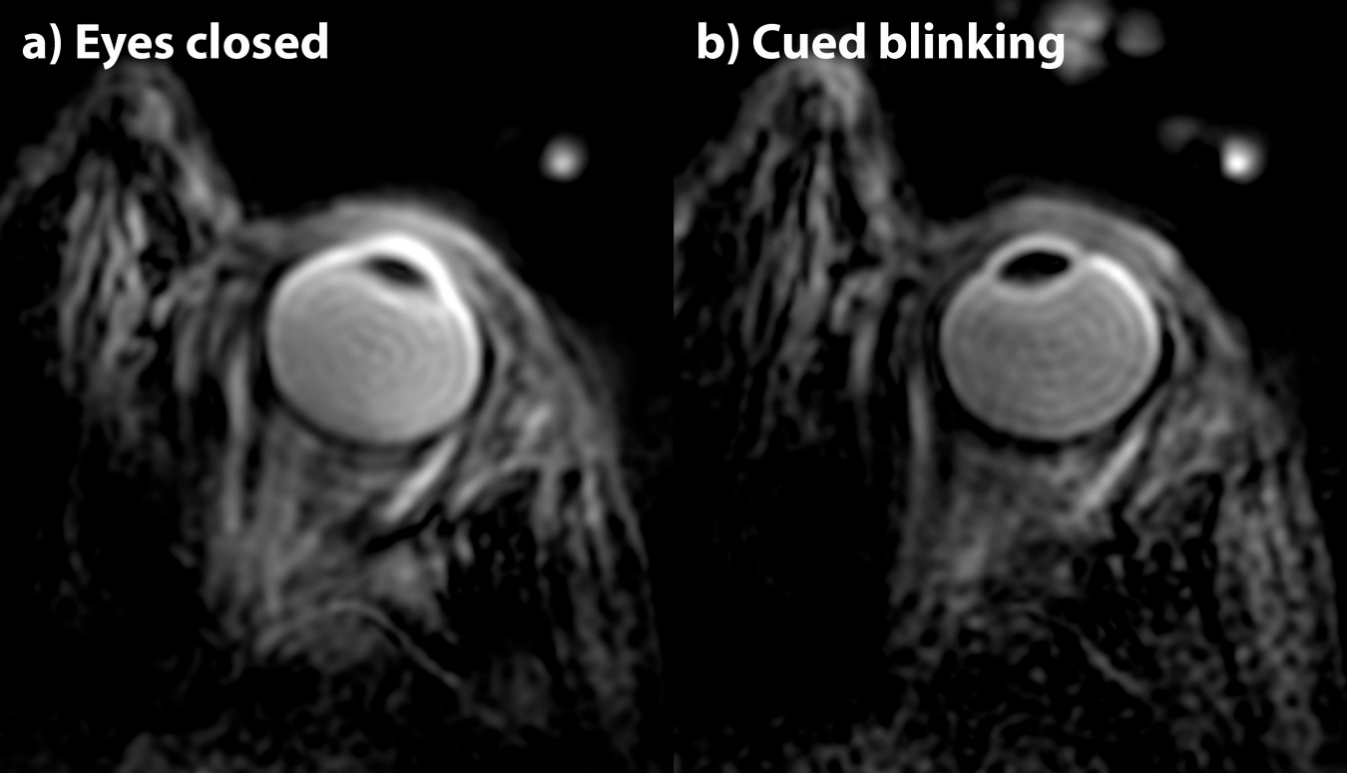
Sensitivity of magnetization-prepared ZTE imaging to eye motion. (a) transverse reformat of a T_2-_prepared ZTE data set obtained in a volunteer instructed to keep the eyes closed during the entire scan. (b) reformat from the same volunteer using the same contrast preparation but during cued-blinking, instructing the volunteer keeping the eye open and focused to a defined spot during the segmented T_2_-prepared ZTE acquisition, with a pause to blink in between segments. No significant difference in image quality was found. The small bright spots on the outside of the head are the capacitors of the receive coil, which are generally not visible in MR-images due to their short T_2_. Although their water-content is small[22], the close proximity to the receive coil results a high signal intensity.

Fig 3 shows results comparing two acquisition regimes to judge the sensitivity of magnetization-prepared ZTE to eye motion. No significant differences in image quality regarding motion induced image artifacts were found between the data obtained during cued-blinking and those when leaving the volunteer with no other instruction other than to keep the eyes closed.

### Patient study

**Fig 4:**
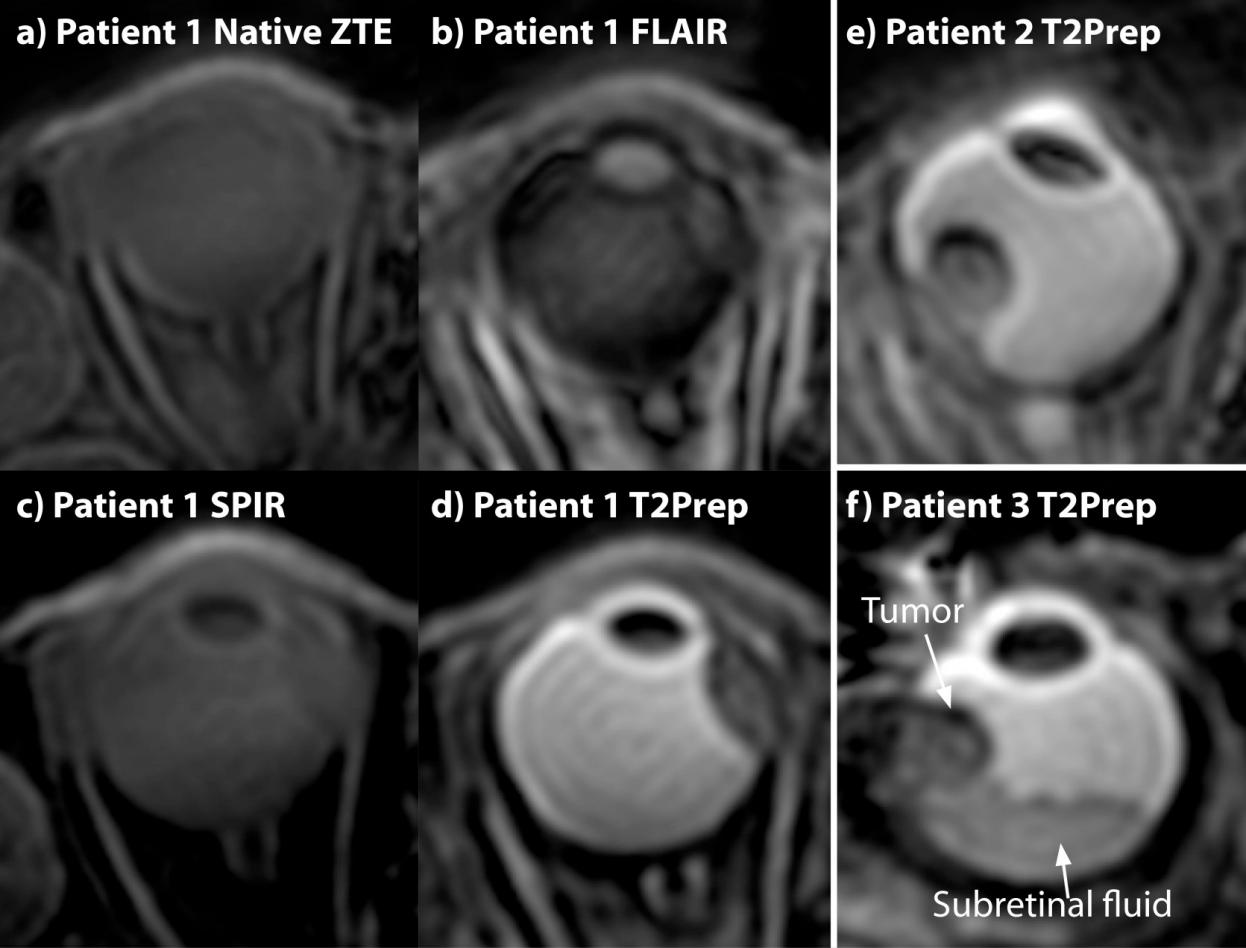
Magnetization-prepared ZTE imaging of the eye of three uveal melanoma patients. (a-d) ZTE images showing different magnetization preparation of one of the patients (isotropic (1mm^3^) resolution). In the native and fat suppressed ZTE the tumor cannot be discriminated from the surrounding tissues. The FLAIR ZTE does result in some degree of contrast between the vitreous body, but the tumor boundaries are not clearly identifiable as not all the signal from the vitreous body is suppressed. The T_2_-prepared ZTE, however, does provide a good contrast in which the tumor boundaries are clearly visible. (e,f) T_2_-prepared ZTE scans of two other patients, showing clearly the tumor in the eye. The third patient (f) furthermore suffers from a retinal detachment, a common complication of an ocular tumor, which results in fluid accumulation behind the retina. This subretinal fluid can be identified as a region which has a signal intensity between the tumor and the vitreous body.

Fig 4 shows a set of images from three different patients included in the pre-study to determine which ZTE-sequence would provide the best image contrast. In the pure ZTE and fat-suppressed ZTE, (Fig 4A and 4C), the tumor cannot be discriminated from the surrounding tissues. However, it can clearly be identified in the FLAIR- and T_2_-prepared ZTE, where in the latter the presence of sub-retinal fluid, a common finding in patients with Uveal Melanoma, can be diagnosed, which was however not visible on the FLAIR-ZTE. Therefore, the T_2_-prepared ZTE was considered to be the favorite contrast for eye tumor diagnosis.

**Fig 5:**
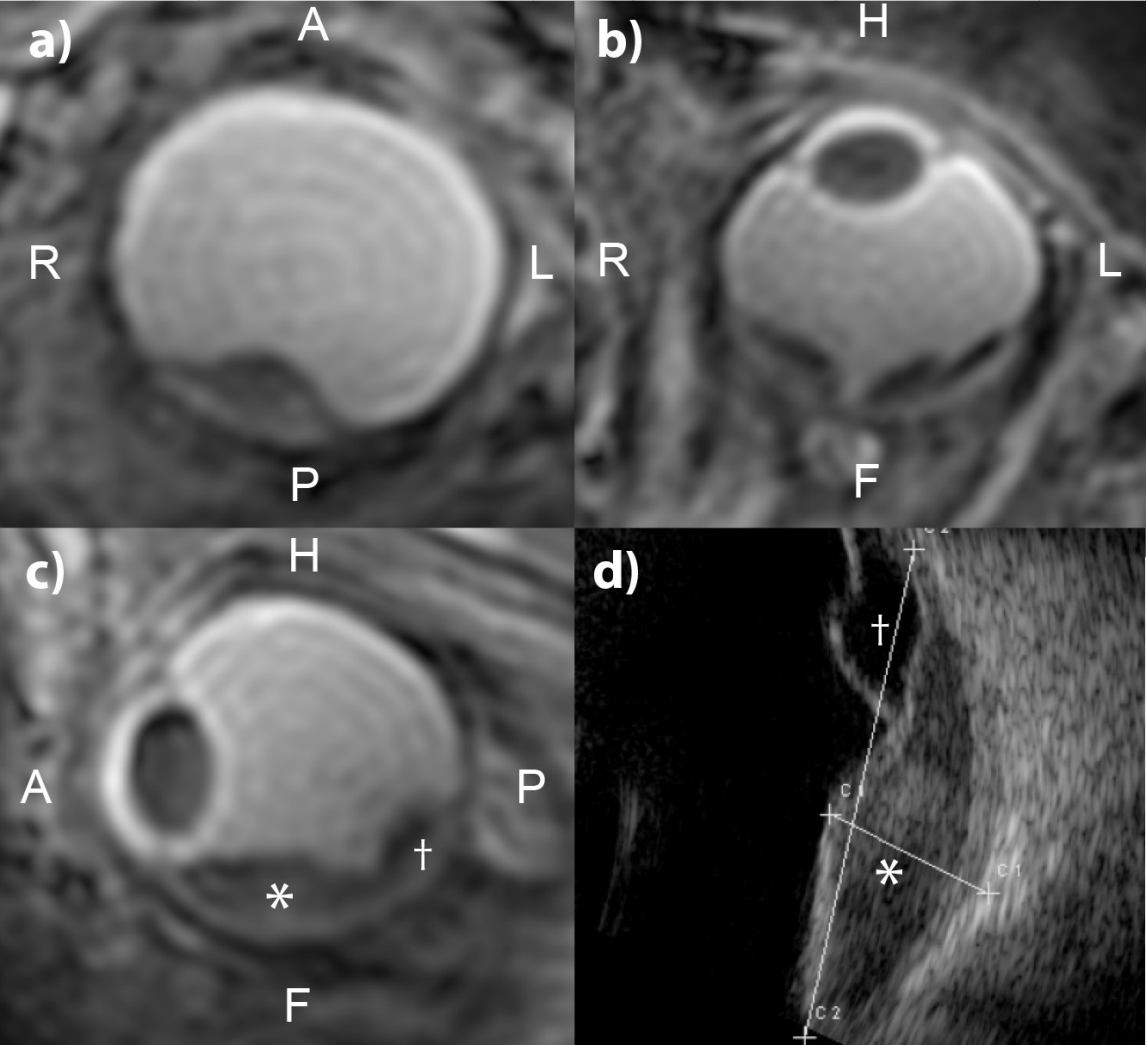
Reformatted high-resolution ZTEs and ultrasound frame for comparison of UM-patient 9. (a,b,c) Reformats of one T_2_-prepared ZTE scan in three orthogonal directions showing the complex three-dimensional shape of the lesion (marked by *) and retinal detachment (marked by †). (d) Ophthalmic ultrasound is only able to provide a 2D cross-section of the lesion (tumor extent marked by c1, c2 lines with crosses).

**Fig 6:**
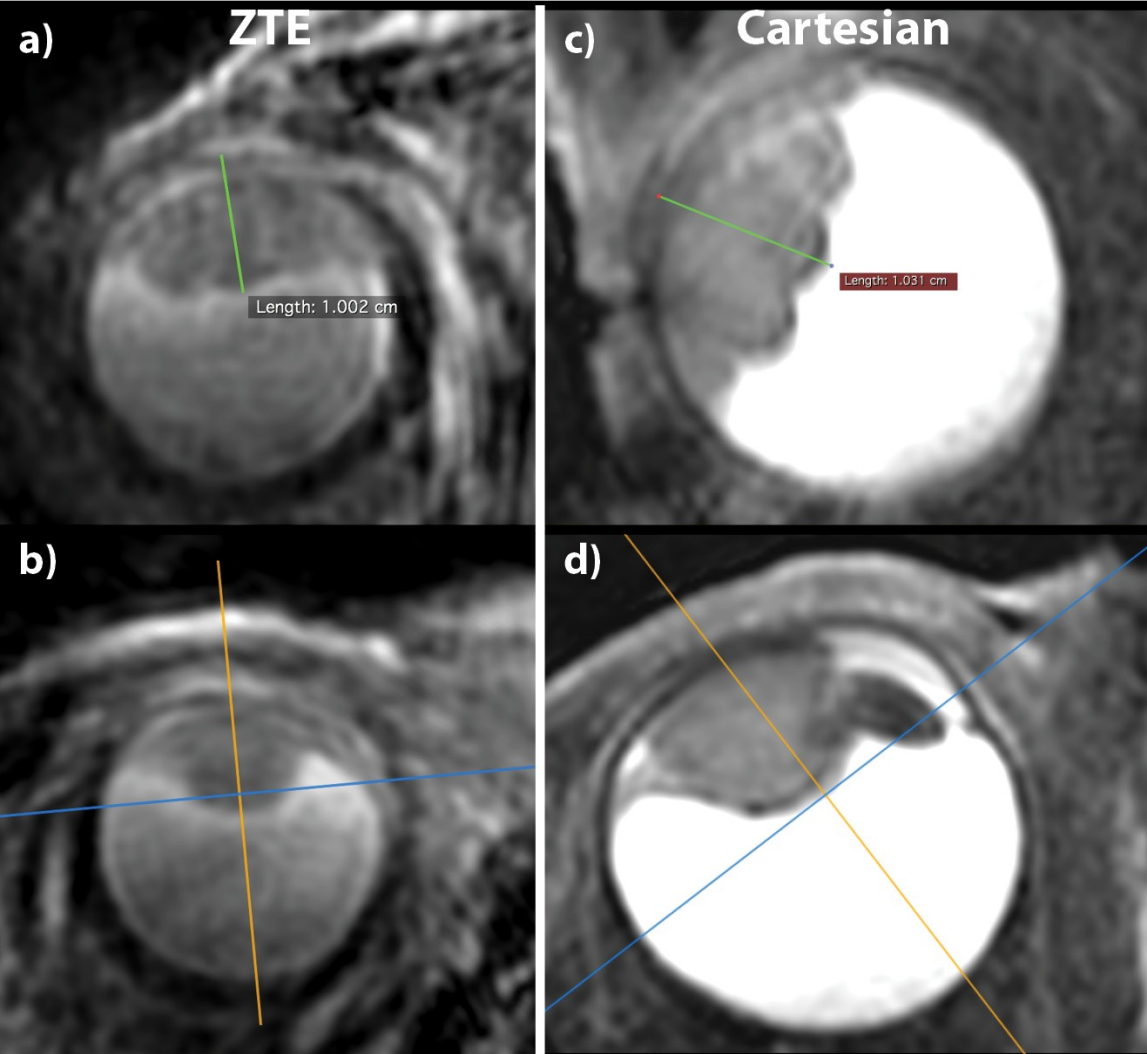
Tumor thickness measurements performed on the ZTE and Cartesian images of UM-patient 7. To measure the tumor thickness, defined as the distance between the top of the tumor and the outside of the sclera, a reformatting of the 3D MR-images is made perpendicular to the tumor (the orange plane in b,d)). On the resulting image, a) and c), the tumor thickness can be accurately measured. The corresponding measures of all patients can be found in Table 3.

In three of the five patients scans with the (0.8mm)^3^ T_2_-prepared ZTE-protocol the images clearly show the tumor and surrounding structures. Fig 5 shows perpendicular reformats in three different directions of one of these patients to demonstrate the value of the isotropic resolution of ZTE. As a comparison the clinical ophthalmic ultrasound is shown, which only provides a single 2D cross-section of the tumor. For these three patients, the tumor thickness measurements performed on the ZTE-scans and on the Cartesian equivalent differ less than the voxel-size, 0.8mm, Table 3 and Fig 6. In one patient (UM6 of Table 3), the contrast between the tumor and vitreous body was low on the ZTE, making it difficult to perform an accurate measurement. As a result, the measured tumor thickness differs 0.8mm compared the measurement on the Cartesian images, in which the tumor could be clearly differentiated. Finally, in one of these patients a low SNR was observed, together with some artifacts that suggest magnetic field inhomogeneities, which made it impossible to delineate the tumor boundaries. Although large signal voids were present in the Cartesian scan of these patients, an accurate measurement was still possible as the artefact was localized more distant from the tumor.

**Table 3:** Comparison of the image quality and the measured tumor thickness on Cartesian and ZTE images.

Patient ID	Cartesian		ZTE	
	Image quality	Tumour thickness	Image quality	Tumor thickness
UM05	5	8.1 mm	4	8.7 mm
UM06	3	4.4 mm	3	5.2 mm
UM07	5	10.3 mm	4	10.0 mm
UM08	4	4.0 mm	1	no valid measurement possible
UM09	5	5.5 mm	4	6.0 mm

For most patients the Cartesian images were of superior diagnostic image quality and scored on a scale from 1 (poor) to 5 (very good) always equal or better compared to the ZTE images. For most patients the difference is less than the voxel-size, except for patient 8 for whom no valid measurement was possible on the ZTE-images due to an insufficient image quality.

### Comparison with Cartesian imaging

**Fig 7:**
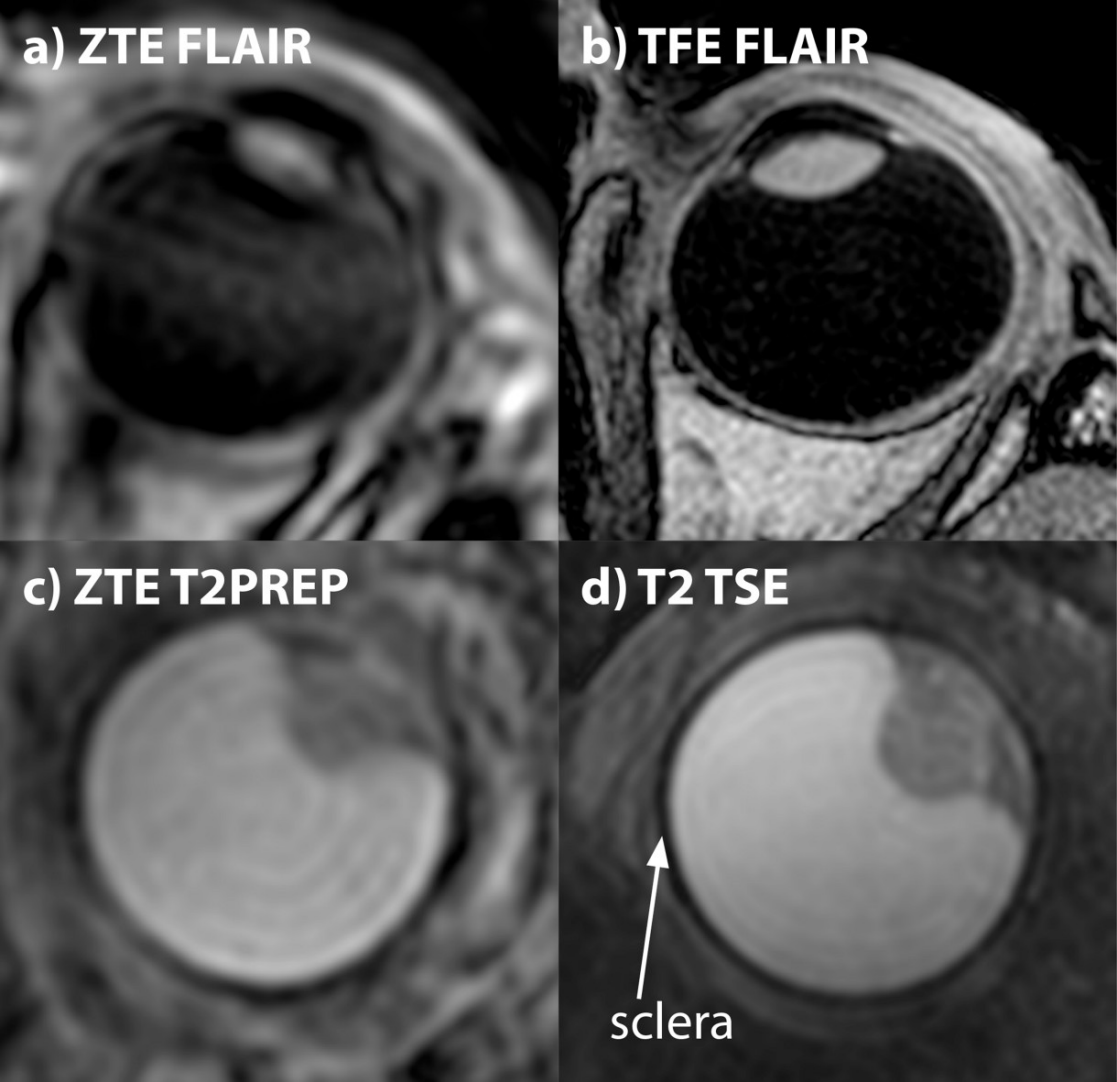
Comparison between magnetization prepared ZTE imaging and conventional gradient echo (TFE) Cartesian imaging. (a,b) Magnetization prepared FLAIR images of a healthy subject, show some contrast washout in the (1mm)^3^ ZTE scan, whereas complete suppression of the vitreous body is achieved in the Cartesian acquisition. (c,d) T_2_-prepared images of UM-patient 5. Although both acquisition methods result in a similar contrast, the higher resolution of the Cartesian imaging allows for a better delineation of the tumor-sclera interface compared to the (0.8mm)^3^ ZTE images.

Fig 7 shows a comparison of the magnetization prepared ZTE-scan and the corresponding Cartesian equivalent. Although the contrast is similar between both acquisition strategies, the effect of the higher resolution of the Cartesian imaging is clearly visible. Furthermore, as all ZTE spokes acquire data from the center of k-space, the rather long echo-trains results in some contrast washout in the ZTE-scan, especially with the FLAIR preparation, which was successfully mitigated in the Cartesian imaging by the use of a low-to-high k-space sampling scheme. Overall, in four of the five patients the Cartesian images provided a higher diagnostic image quality than their T_2_-prepared ZTE equivalent, Table 3.

All volunteers and patients reported a much more comfortable experience during the ZTE scans than during their Cartesian equivalents, with much less propensity to blink due to the almost silent nature of the scans. All ZTE scans were approximately 10-20dB quieter than their Cartesian equivalent, Table 4. The sound pressure of native ZTE was 73dB, whereas the maximum sound pressure of the T_2_-prepared ZTE was 82dB. This increase in sound pressure was caused by the fast gradient switching of crusher gradients in the preparation module. The subjective experience of sound level of these magnetization prepared ZTE scans, was however similar to the native ZTE scan, as this gradient switching only produces a very short sound. This can be reduced in future implementations by using more appropriate crusher gradients, which might, however, slightly increase the scan time as these optimized crushers might need more time for a similar amount of signal suppression. For all Cartesian scans, the sound pressure was approximately 93dB, as the sound pressure was predominantly caused by the switching readout gradients. The relatively long duration of these sounds, at least half a second depending on the read-out scheme, made the subjective experience less comfortable than the short clicks, of only a few milliseconds, of the T_2_-preparation module of the ZTE acquisition.

**Table 4:** Comparison of the highest registered acoustic sound pressure of the Cartesian and ZTE scans.

	ZTE sound pressure (dB)	Cartesian sound pressure (dB)
Native	73	93
FLAIR	78	95
SPIR	77	
SAT	73	
T2-prepared	82	93

The relatively high maximal sound pressure of the T_2_-prepared ZTE was caused by the crusher gradients of the preparation module. These were audible as small short clicks in a scan which was experienced as equally silent as the native ZTE scan.

## Discussion

Although native proton-density weighted ZTE MR-images provide insufficient contrast in the human eye, magnetization-prepared ZTE allows for sufficient contrast and flexibility for ocular imaging. The SPIR-prepared ZTE is not particularly useful in measuring tumor size due to lack of contrast, but it could be useful for investigating diseases of the optic nerve since the combination of SPIR and ZTE gives good visualization of the optic nerve at rather high temporal sampling efficiency. The MT-effect, associated with the applications of off-resonant RF pulses, also introduces contrast between the lens and the vitreous body. The T_2_-prepared ZTE gives the highest contrast. In addition, there is significant contrast between the vitreous body and subretinal fluid. Although the FLAIR is yielding one of the essential contrasts in Cartesian eye imaging, the resulting scanning time for the FLAIR ZTE is unacceptably long. Furthermore, as every read-out starts in the center of k-space, the ZTE FLAIR has some pronounced contrast washout, which is not present in the Cartesian FLAIR due to the low-to-high k-space sampling scheme. The saturation prepared ZTE, which results in a similar contrast to FLAIR ZTE due to the long T1 of the vitreous body, partly resolves these limitations as the magnetization preparation pulses can be repeated every 900 ms instead of every 3 seconds for the FLAIR ZTE. This reduces the scan time from 6 to less than 2 minutes and allows for a short echo-train to minimize the contrast washout. The saturation pulses do, however, suppress most of the MR-signal, which results in a lower SNR.

As the ZTE uses 3D radial FID-sampling, no spatial localized RF-excitation pulses can be used, making the technique intrinsically susceptible to aliasing artefacts stemming from outside the FOV. The use of a local receive coil limits the spatial receive sensitivity to the eye and surrounding tissues, allowing for a FOV of roughly 120 mm without fold-over artifacts. Although for bilateral eye imaging an increased FOV would be needed, this should not necessarily lead to increased scanning times since for both eyes an individual receive coil would be needed, which would allow for the use of under-sampling techniques such as SENSE or GRAPPA. [23,24]

The main advantage of ZTE eye imaging compared to conventional Cartesian imaging is the increased patient comfort due to its silent nature which all patients preferred. The robustness of the ZTE against eye-motion (due to oversampling the centre of k-space) makes it a robust alternative for cued-blinking paradigms. Claustrophobic patients or very nervous subjects often fail to adhere to the cued-blinking instructions which results in significant motion artefacts, that may mask clinically relevant details in the MR-images[25]. For these patients a silent MRI-exam, in which they can keep their eyes closed, could be the only method to acquire the clinically needed information on tumor size and location. Although a bias might have been introduced by the non-randomized ordering of the uninstructed and cued-blinking instructed ZTE-scans, the lack of motion artefacts in the uninstructed patient scans shows the motion robustness of the ZTE-technique against eye-motion. It should be noted that without explicit eye focusing the localization or the orientation of the tumor with respect to the main optical axis is not precisely defined, which means that post processing of the ZTE data would be necessary to define this axis[3]. This can be facilitated due to the isotropic spatial resolution. Reformatting in any desired orientation is possible after scanning allowing a corresponding inspection and a simplified workflow for treatment planning.

Although this pilot study included only a limited number of patients and not all sequences have been tested in all patients due to time constraints, it shows the potential strengths but also the weaknesses of ZTE in eye applications. The initial ZTE scans to determine the optimal contrast used a conservative 1 mm^3^ resolution. Although this resolution is sufficient to show how different contrasts can be achieved in eye ZTE-scanning trough magnetization preparation, the significant partial volume effects hinder clinical interpretation of these images. The sclera could, for example, not always be identified as it is on average only 0.5 mm thick. The ZTE scans with a higher resolution on (0.8mm)^3^ mitigated this issue up to a certain extent, but some ringing artifacts still mask most fine anatomical details. However, as these scans do show sufficient detail to determine the tumor dimensions, they can be used to determine the optimal treatment strategy for these patients, [4] which mainly depends on the tumor thickness and basal diameter. For the majority of the included patients the ZTE-images showed a similar tumor thickness compared to the conventional Cartesian images, although in one patient the image quality of the ZTE was too low for clinical evaluation.

For additional, more comprehensive, evaluations, such as the screening for scleral invasion of the tumor, an increased resolution compared to the current ZTE protocol will be needed. Given the large FOV needed for the ZTE, such a scan would take at least six minutes to acquire a sufficient number of spokes, if not more, and to achieve a decent SNR. Such long scan times make the scan very susceptible to slow drifts in gazing direction [26] which blur out the fine anatomical details one aims to resolve using the high resolution. We therefore expect that a significant improvement of image resolution will not be easily possible, although finally in vivo measurements are needed to definitively confirm this. An increased resolution could, however, also help mitigating the Gibbs-like ringing artifacts, which are potentially caused by the low matrix size. It remains thus to be determined by future work if magnetization prepared ZTE can provide such high resolution, as the available SNR is limited due the relatively low flip angle and the potentially rather long scan times might be in conflict with patient tolerance.

Additionally, the specific magnetization preparation blocks used for ZTE need attention with respect to image quality. As the eye is surrounded by air, not only anteriorly but also in the nasal cavities, and bony structures of the globe, local field inhomogeneities remain present, even after localized shimming. These B_0_ inhomogeneities can for instance influence the T_2-_preparation module, as off-resonance effects can compromise the performance of the adiabatic RF pulses employed, due to their bandwidth restrictions. Also here further work is needed in redesigning those blocks, especially if these techniques will be used in the planning of proton therapy, since the tantalum markers used as an anatomical reference, will increase local B_0_ inhomogeneities.

However, as the ZTE acquisition does not need a regular pause for the cued-blinking paradigm, the ZTE protocols can acquire more profiles in the same amount of time compared to the Cartesian acquisition, which can partly compensate its intrinsic lower MR-signal strength at the cost of slight contrast changes.

Given the combination of the relatively low SNR of the ZTE-technique and the relatively small anatomy of the eye, these evaluations were performed at 7 Tesla MRI to benefit from the increased SNR of high field MRI. Recent advances in MRI have made high quality ocular MRI feasible at clinical 3 Tesla, although at a slightly lower resolution than at 7 Tesla[26]. Since these evaluations are relatively field strength independent, a comparable evaluation at 3T could reach similar results.

## Conclusion

Magnetization-prepared ZTE imaging of the eye at 7T provides images with good contrast in both healthy subjects and patients with an intra-ocular tumor. Compared to the conventional Cartesian acquisition strategies, the resolution and image quality of the current ZTE protocol is a limiting factor, while its robustness against eye-motion and increased patient comfort are clearly advantageous. As the current ZTE protocol allows for a basic assessment of the tumor dimensions, this protocol could be advantageous for patients who do not comply with regular scan protocols due the high sound pressure of conventional MR-imaging. However, in other cases, the conventional Cartesian scanning is preferred as the higher image quality / resolution provides more diagnostic value.

## References

[1] de Graaf P, Göricke S, Rodjan F, Galluzzi P, Maeder P, Castelijns JA, et al Guidelines for imaging retinoblastoma: imaging principles and MRI standardization. Pediatr Radiol. Springer-Verlag; 2012;42(1):2–14. 10.1007/s00247-011-2201-5 21850471 PMC3256324

[2] Diogo MC, Jager MJ, Ferreira TA. CT and MR Imaging in the Diagnosis of Scleritis. AJNR Am J Neuroradiol. 2016 12;37(12):2334–9. 10.3174/ajnr.A4890 27444937 PMC7963878

[3] Beenakker J-WM, Shamonin DP, Webb AG, Luyten GPM, Stoel BC. Automated retinal topographic maps measured with magnetic resonance imaging. Invest Ophthalmol Vis Sci. 2015 2;56(2):1033–9. 10.1167/iovs.14-15161 25593030

[4] Beenakker J-WM, Ferreira TA, Soemarwoto KP, Genders SW, Teeuwisse WM, Webb AG, et al Clinical evaluation of ultra-high-field MRI for three-dimensional visualisation of tumour size in uveal melanoma patients, with direct relevance to treatment planning. Magn Reson Mater Phy. 2016 6;29(3):571–7. 10.1007/s10334-016-0529-4 PMC4891368 26915081

[5] Graessl A, Muhle M, Schwerter M, Rieger J, Oezerdem C, Santoro D, et al Ophthalmic magnetic resonance imaging at 7 T using a 6-channel transceiver radiofrequency coil array in healthy subjects and patients with intraocular masses. Invest Radiol. 2014 5;49(5):260–70. 10.1097/RLI.0000000000000049 24651662

[6] Berkowitz B, Detroit M, Canfield D, McDonald C, Ito Y, Tofts P, et al Measuring the human retinal oxygenation response to a hyperoxic challenge using MRI: Eliminating blinking artifacts and demonstrating proof of concept. Magn Reson Med. John Wiley & Sons, Inc; 2001 8 1;46(2):412–6. 10.1002/mrm.1206 11477648

[7] Beenakker JWM, van Rijn GA, Luyten GPM, Webb AG. High-resolution MRI of uveal melanoma using a microcoil phased array at 7 T. NMR Biomed [Internet]. 2013 12;26(12):1864–9. Available from: 10.1002/nbm.3041 24123279

[8] Hafner S. Fast imaging in liquids and solids with the Back-projection Low Angle ShoT (BLAST) technique. Magn Reson Imaging. 1994;12(7):1047–51. 10.1016/0730-725x(94)91236-p 7997092

[9] Weiger M, Brunner DO, Dietrich BE, Müller CF, Pruessmann KP. ZTE imaging in humans. Magn Reson Med. 2013 8;70(2):328–32. 10.1002/mrm.24816 23776142

[10] Schieban K, Weiger M, Hennel F, Boss A, Pruessmann KP. ZTE imaging with enhanced flip angle using modulated excitation. Magn Reson Med. 2015 9;74(3):684–93. 10.1002/mrm.25464 25242318

[11] Larson PEZ, Han M, Krug R, Jakary A, Nelson SJ, Vigneron DB, et al Ultrashort echo time and zero echo time MRI at 7T. Magn Reson Mater Phy. Springer Berlin Heidelberg; 2016 6;29(3):359–70. 10.1007/s10334-015-0509-0 PMC4892974 26702940

[12] Börnert P, Stehning C, Nehrke K, Groen J, Webb AG. Using high permittivity materials to improve ZTE image quality at 7T. 2015 p. 55.

[13] Wong ST, Roos MS. A strategy for sampling on a sphere applied to 3D selective RF pulse design. Magn Reson Med. Wiley Subscription Services, Inc., A Wiley Company; 1994 12;32(6):778–84. 10.1002/mrm.1910320614 7869901

[14] Wu Y, Ackerman JL, Chesler DA, Li J, Neer RM, Wang J, et al Evaluation of Bone Mineral Density Using Three-Dimensional Solid State Phosphorus-31 NMR Projection Imaging. Calcif Tissue Int. Springer-Verlag; 1998;62(6):512–8. 10.1007/s002239900471 9576979

[15] Dannels WR. Magnetic resonance imaging with short echo times. US Patent Office; 2009 Nov 24.

[16] Brittain JH, Hu BS, Wright GA, Meyer CH, Macovski A, Nishimura DG. Coronary Angiography with Magnetization‐Prepared T2 Contrast. Magn Reson Med. Wiley Subscription Services, Inc., A Wiley Company; 1995 5 1;33(5):689–96. 10.1002/mrm.1910330515 7596274

[17] Balaban R, Ceckler T. Magnetization transfer contrast in magnetic resonance imaging. Magn Reson Quart. 1992;8(2):116–37. 1622774

[18] Richdale K, Sinnott LT, Bullimore MA, Wassenaar PA, Schmalbrock P, Kao C-Y, et al Quantification of age-related and per diopter accommodative changes of the lens and ciliary muscle in the emmetropic human eye. Invest Ophthalmol Vis Sci. The Association for Research in Vision and Ophthalmology; 2013 2;54(2):1095–105. 10.1167/iovs.12-10619 23287789 PMC3567753

[19] Richdale K, Wassenaar P, Teal Bluestein K, Abduljalil A, Christoforidis JA, Lanz T, et al 7 Tesla MR imaging of the human eye in vivo. J Magn Reson Imaging. 2009 11;30(5):924–32. 10.1002/jmri.21959 19856406 PMC2871321

[20] Jones CE, Pope JM. Measuring optical properties of an eye lens using magnetic resonance imaging. Magn Reson Imaging. 2004 2;22(2):211–20. 10.1016/j.mri.2003.07.005 15010113

[21] Robson MD, Bydder GM. Clinical ultrashort echo time imaging of bone and other connective tissues. NMR Biomed. John Wiley & Sons, Ltd; 2006 11 1;19(7):765–80. 10.1002/nbm.1100 17075960

[22] Horch RA, Wilkens K, Gochberg DF, Does MD. RF coil considerations for short-T2 MRI. Magn Reson Med. 2010 7 27;64(6):1652–7. 10.1002/mrm.22558 20665825 PMC2970722

[23] Griswold MA, Jakob PM, Heidemann RM, Nittka M, Jellus V, Wang J, et al Generalized autocalibrating partially parallel acquisitions (GRAPPA). Magn Reson Med. John Wiley & Sons, Ltd; 2002 6;47(6):1202–10. 10.1002/mrm.10171 12111967

[24] Pruessmann KP, Weiger M, Scheidegger MB, Boesiger P. SENSE: sensitivity encoding for fast MRI. Magn Reson Med. 1999 11;42(5):952–62. 10542355

[25] Wezel J, Garpebring A, Webb AG, van Osch MJP, Beenakker J-WM. Automated eye blink detection and correction method for clinical MR eye imaging. Magn Reson Med. 2017 7;78(1):165–71. 10.1002/mrm.26355 27476861

[26] Ferreira TA, Grech Fonk L, Jaarsma-Coes MG, van Haren GGR, Marinkovic M, Beenakker J-WM. MRI of Uveal Melanoma. Cancers (Basel). Multidisciplinary Digital Publishing Institute; 2019 3 17;11(3):377. 10.3390/cancers11030377 PMC6468484 30884881

